# Partnering With Community Institutions to Increase Access to Healthful Foods Across Municipalities

**DOI:** 10.5888/pcd10.130011

**Published:** 2013-10-03

**Authors:** Lara Jaskiewicz, Rachael D. Dombrowski, Heather M. Drummond, Gina Massuda Barnett, Maryann Mason, Christina Welter

**Affiliations:** Author Affiliations: Rachael D. Dombrowski, Chicago Public Schools, Chicago, Illinois; Heather M. Drummond, Christina Welter, University of Illinois Chicago, Chicago, Illinois; Gina Massuda Barnett, Cook County Department of Public Health, Oak Forest, Illinois; Maryann Mason, Department of Pediatrics, Feinberg School of Medicine, Northwestern University, Chicago, Illinois.

## Abstract

**Background:**

Low-income and minority communities have higher rates of nutrition-related chronic diseases than do high-income and nonminority communities and often have reduced availability to healthful foods. Corner store initiatives have been proposed as a strategy to improve access to healthful foods in these communities, yet few studies evaluating these initiatives have been published.

**Community Context:**

Suburban Cook County, Illinois, encompasses 125 municipalities with a population of more than 2 million. From 2000 through 2009, the percentage of low-income suburban Cook County residents increased 41%; African-American populations increased 20%, and Hispanic populations increased 44%. A 2012 report found that access to stores selling healthful foods was low in several areas of the county.

**Methods:**

Beginning in March 2011, the Cook County Department of Public Health recruited community institutions (ie, local governments, nonprofit organizations, faith-based institutions) who recruited corner stores to participate in the initiative. Corner stores were asked to add new, healthful foods (May–June 2011) to become eligible to receive new equipment, marketing materials, and enhanced community outreach (July 2011–February 2012).

**Outcomes:**

Nine community institutions participated. Of the 53 corner stores approached, 25 (47%) participated in the trial phase, which included offering 6 healthful foods in their stores. Of those, 21 (84%) completed the conversion phase, which included expansion of healthful foods through additional equipment and marketing and promotional activities.

**Interpretation:**

Community institutions can play a key role in identifying and engaging corner stores across jurisdictions that are willing and able to implement a retail environment initiative to improve access to healthful foods in their communities.

## Background

Unhealthful eating and sedentary lifestyles contribute to many negative health outcomes, including the development of chronic conditions such as diabetes, heart disease, and stroke (1,2). The rising incidence of these diseases continues to strain our health care system (3). In the United States, nearly 24 million people have limited or no access to healthful foods (4).

Communities of low socioeconomic status are more likely than communities of high socioeconomic status to have fewer stores selling fresh produce or to have to travel farther to reach such a store (4,5). These low-access communities generally have many fast-food restaurants, chain pharmacies, and other small food stores, while having few full-service grocery stores (6–8).

This phenomenon has spurred initiatives to encourage small grocery stores (corner stores) in low-access communities to sell more healthful foods (9–11). Because such initiatives are new, few reports exist describing methods for community engagement and factors influencing success. This article contributes to the knowledge base for implementation of corner store initiatives in multiple communities.

### Community Context

Suburban Cook County surrounds the city of Chicago, Illinois, in the Midwestern United States. It covers 735 square miles and encompasses 125 municipalities with 2,233,179 people (12). Similar to nationwide trends, the population living at or below 200% of the federal poverty guidelines in suburban Cook County increased 41% from 2000 through 2009 (12–14); the African American population increased 20%, and the Hispanic population increased 44% (12–14).

Minority populations are disproportionately affected by chronic diseases in suburban Cook County (14). For example, the rate of coronary heart disease among African Americans (152.8 per 100,000 population) is 17% higher than the rate for whites, and 52% higher than the Healthy People 2020 goal of 100.8 per 100,000 population (15). The diabetes mortality rate for African Americans in suburban Cook County (93.5 per 100,000 population) is 85% higher than the rate for whites.

**Figure 1:**
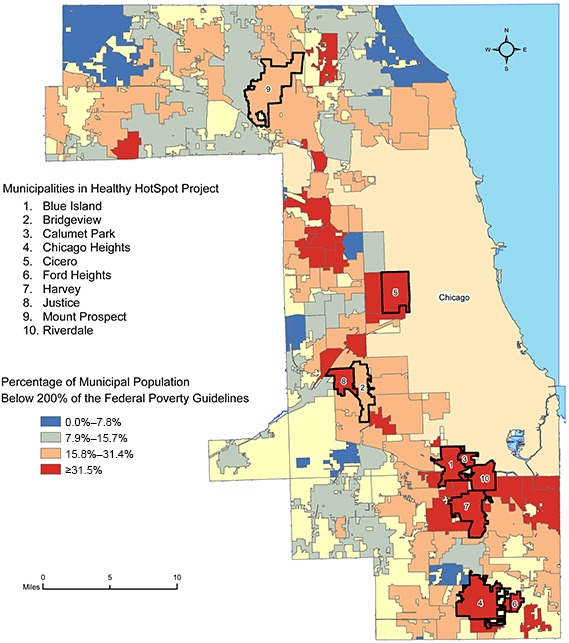
Population below 200% of the federal poverty guidelines, Healthy HotSpot Corner Store Initiative, Suburban Cook County, Illinois, 2005–2009 (13). Map created by the Cook County Department of Public Health, Epidemiology Unit. Abbreviation: FPG, federal poverty guidelines.

Some of the nation’s poorest communities are in suburban Cook County (Figure 1), and chronic disease mortality rates there are the highest in the Midwest. Furthermore, capacity to deliver human services varies widely across the county, and municipalities with the greatest needs often have the lowest capacity to address them (16). Lack of infrastructure and coordination of services also continuously contributes to health inequities in suburban Cook County (16).

In 2010, the Cook County Department of Public Health (CCDPH) identified municipalities in suburban Cook County with limited access to healthful foods (6) and designed a corner store initiative that applied community engagement principles and leveraged trust of local community institutions. This approach aimed to promote community ownership, initiative sustainability, and community promotion of availability of healthful foods to increase consumer demand for the sale of these foods. Outcomes of interest were 1) increased capacity of community institutions to work with local stores, and 2) increased corner store owner capacity to identify, stock, and sell more healthful foods.

## Methods

### Intervention

In collaboration with the Public Health Institute of Metropolitan Chicago, CCDPH used funding from Communities Putting Prevention to Work (CPPW) to implement a corner store initiative, branded as “Healthy HotSpot.” The location included multiple municipalities in suburban Cook County. This initiative was 1 component of the CPPW initiative in suburban Cook County, which focused on changing policies, systems, and environments to prevent obesity by promoting healthful eating and active living. The national CPPW initiative focused on both obesity prevention and tobacco control, with the goal of reducing the burden of chronic disease (10).

**Figure 2:**
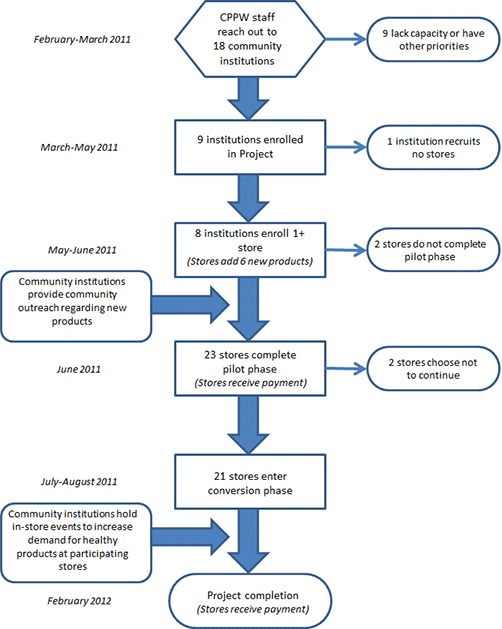
Recruitment process of the Healthy HotSpot Corner Store Initiative, Suburban Cook County, Illinois, 2011–2012.

Based on a model developed by The Food Trust in Philadelphia, Pennsylvania, the Healthy HotSpot initiative partnered with community institutions to increase sales of healthful foods in local corner stores. Unlike most corner store initiatives (which include 1 partner working with many stores), Healthy HotSpot worked with 8 separate community institutions to reach 21 stores in various suburban Cook County communities (17). Healthy HotSpot staff received training on healthy corner store initiatives and provided input to the Healthy HotSpot initiative plan on the basis of their suburban Cook County experiences. The Healthy HotSpot initiative began with a trial phase, with store recruitment and the addition of new foods that met program criteria. Staff notified store owners that if they successfully added the new foods, they would receive $250 and be invited to participate in the second phase (conversion). The conversion phase provided stores with new equipment, marketing materials, enhanced community outreach and engagement, and an additional $250 (Figure 2).

### Community engagement

Local community institutions (ie, local governments, nonprofits, faith-based institutions) became integral partners in the initiative because of detailed community knowledge and local presence, which assisted in determining candidate stores and allowed for regular interaction with store owners. Local institutions also served as representatives of the initiative in each municipality and conducted community promotion for increased consumption of healthful foods.

The initiative started in March 2011, with a planned ending in February 2012. Several months before the initiative began Healthy HotSpot staff conducted community outreach within suburban Cook County to solicit applications for a local grant program. Municipalities with a high percentage of low-income residents were a focus. When the corner store initiative was ready to begin, Healthy HotSpot staff contacted community institutions from those primarily low-income communities that had expressed concerns about food access while ensuring regional participation (Figure 1). Community institution recruitment occurred in late March and early April 2011.

To build the capacity of community institutions, Healthy HotSpot staff conducted a day-long store recruitment workshop and 3 technical assistance webinars. The workshop occurred before store recruitment and explained the initiative structure and how to build relationships with corner store owners. Subsequent webinars provided guidance on community outreach and marketing and on pricing, purchasing, and placement of healthful foods. The webinars formed the basis of fact sheets to distribute to store owners.

Community institutions were responsible for identifying and recruiting candidate local corner stores into the initiative during late April and early May 2011. Candidate stores had small product selections, sold food, and had 1 checkout counter. Community institutions had authority in identifying the number and characteristics of candidate stores (eg, stores serving a specific neighborhood). If stores agreed to participate in the trial phase, the community institution notified its designated Healthy HotSpot staff person, who visited the store with a representative of the community institution. During their visit, this pair completed a baseline store product assessment and enrollment form (based on The Food Trust’s protocol). Store owners received materials including an initiative overview and a healthy product menu, in English or Spanish (www.healthyhotspot.org).

Store owners committed to add 6 new foods, including 1 fresh fruit, 1 fresh vegetable, and 4 foods chosen from additional categories — low-fat dairy, lean proteins, canned or frozen fruits and vegetables, or whole grains. Stores used existing equipment to store foods. After the store added the new foods (within 4 to 6 weeks), Healthy HotSpot and community institution staff visited the store together to document the new product additions, provide successful owners with a $250 check, and enroll the store in the conversion phase. The trial phase was scheduled to last from mid-May to late June 2011.

The conversion phase consisted of providing new equipment, increasing marketing, and stores continuing to sell healthful foods. Healthy HotSpot staff purchased equipment from grocery industry suppliers and met county environmental health standards. Municipal health inspectors received letters to inform them of the initiative and provide an avenue for questions or concerns. None were raised.

Two Healthy HotSpot staff members led the conversion process, dividing up the stores between them. They visited each store with community institution staff and assessed the store’s needs for equipment including refrigerators, freezers, shelving, display baskets, and scales. On the basis of discussion with the store owner, a list of the new equipment was developed for each store that included the location of the equipment in the store. Healthy HotSpot staff shared the equipment plans with store owners to ensure agreement before new equipment was purchased and to show the local health inspector. The conversions cost an average of $3,500 per store. The conversion phase was to begin by mid-July 2011 and last through February 2012. Healthy Hotspot staff notified community institution staff of large equipment delivery dates, and they participated as their schedules allowed. Conversion visits were the only times Healthy HotSpot staff interacted directly with the store owners without the presence of the community institution.

The initiative developed and provided marketing materials for the stores participating in the conversion phase. In-store materials included posters, shelf tags, stickers, and end-of-aisle flags. The intention was to draw shoppers’ attention to the healthful foods in the stores; community institution staff helped to place these items. The community institutions conducted regular community outreach about store changes through newsletters, flyers at community events, or news articles. They were expected to host an in-store event, such as a taste test, in each store.

The Healthy HotSpot initiative had a $200,000 budget for store incentive payments, store equipment, and community institution resources. A separate budget paid for Healthy HotSpot staff time and initiative marketing materials. Healthy HotSpot and CCDPH staff developed the marketing materials in-house and used commercial printers for the final product.

### Evaluation

The process evaluation took place during April and May 2012, in parallel with a program evaluation. The program evaluation included interviews with community institutions and store owners. We report the results of the process evaluation, which used quantitative analysis of data from existing initiative and communications records; therefore, our study protocol was not submitted for human subjects review. The initiative implementation plan laid out the expected steps, actors, and timing. Relevant initiative records included staff e-mails, store enrollment and follow-up assessment forms, training attendance records, outreach plans and reports, memoranda of understanding between the Healthy HotSpot initiative and community institutions and between the Healthy HotSpot initiative and participating stores, and other initiative documentation. The process evaluation asked the following questions:

- Fidelity of implementation: How closely did the initiative implementation follow the initiative plan?
- Dose delivered: What types of training were provided to participating community institutions? Which community institutions participated in trainings?
- Dose received: Did community partners successfully recruit stores into the initiative? Did participating stores increase the number of healthful foods offered?
- Recruitment: What planned and actual procedures were used by Healthy HotSpot staff to recruit community institutions? What planned and actual procedures were used by community partners to recruit stores?
- Context: What external factors affected implementation?

## Outcomes

Sixteen community institutions were identified as potential partners by Healthy HotSpot staff on the basis of their location in a low-income community or their previously stated interest in community food access. As planned, they were contacted through e-mail and telephone. Of those, 7 were interested and believed they had the staff available to participate in the initiative. An additional 2 institutions were recruited in high-need communities, 1 of which joined after the start of the trial phase (Table 1). Thus, 9 community institutions participated in the initiative, 8 of which attended a mandatory workshop on the initiative model. The community institutions comprised 3 municipal governments, 4 nonprofit organizations, and 2 faith-based institutions (Table 2).

**Table 1:** Municipality Demographics, Healthy HotSpot Corner Store Initiative, Suburban Cook County, Illinois, 2011–2012^a^

Municipality	Population	% Below 200% Federal Poverty Guidelines	% Black	% Latino
Blue Island	23,706	43.7	30.8	47.0
Bridgeview and Justice	29,372	32.1	11.6	14.2
Calumet Park	7,835	38.3	88.3	6.8
Chicago Heights	30,276	48.8	41.5	33.9
Cicero	83,891	49.8	3.8	86.6
Ford Heights	2,763	61.9	95.6	1.5
Harvey	25,282	56.4	75.8	19.0
Mount Prospect	54,167	18.0	2.4	15.5
Riverdale	13,549	46.0	93.7	1.7

a Data obtained from the US Census Bureau (13).

**Table 2:** Characteristics and Achievements of Municipality and Community Institutions That Participated in Health HotSpot Corner Store Initiative, Suburban Cook County, Illinois, 2011–2012

Municipality	Institution type	Stores Approached	Trial Phase Stores	Conversion Phase Stores	Conversion Rate, %^a^
Blue Island^b^	Government	6	3	2	67^b^
Bridgeview and Justice	Faith-based	4	0	0	NA
Calumet Park	Nonprofit	6	2	1	50
Chicago Heights	Nonprofit	8	2	2	100
Cicero^b^	Nonprofit	9	8	7	88^b^
Ford Heights	Faith-based	2	2	2	100
Harvey	Nonprofit	10	4	3	75
Mount Prospect	Government	4	1	1	100
Riverdale	Government	4	3	3	100
Total	NA	53	25	21	84

Abbreviation: NA, not applicable.

a The percentage of pilot phase stores that became conversion phase stores.

b Municipalities in which some stores experienced an accelerated trial phase.

In the spring of 2011, representatives from each of the 9 community institutions visited local corner stores (mean, 5.9 stores; Table 2) to meet in person with the owners. Although community institutions were expected to recruit stores independently, only 1 community institution did not need Healthy HotSpot staff support. Healthy HotSpot staff participated in multiple recruitment visits, even in cases in which a relationship existed with the owner. Eight institutions successfully recruited at least 1 store into the trial phase.

Twenty-five stores were initially recruited into the initiative. Of these, 2 did not complete the trial phase. Two stores that completed the trial phase opted out of the conversion phase. One was sold to a new owner planning to convert it to a liquor store. The other closed due to economic reasons. At the transition from trial phase to conversion phase, 2 community institutions requested the addition of new stores, which were brought into the initiative with an accelerated trial timeline. Each store that completed the trial phase was asked to participate in the conversion phase. Of 53 stores approached, 25 (47%) participated in the trial phase. Of these, 21 (84%) completed the conversion phase (Table 2).

In addition to the required fresh fruits and vegetables, the most commonly added food type was whole grains (15 stores). Low-fat dairy products were added in 10 stores and canned fruits or vegetables in 9 stores. The least popular category was lean protein.

Over 7 months, the 8 community institutions participated in an average of 1.9 training sessions of the 4 offered (range 0–4). The highest rate of participation was for the initial in-person orientation to the Healthy HotSpot initiative. Only 3 institutions participated in the final webinar, which focused on increasing consumer demand and promotion of participating stores. During the initiative, half of the community institutions experienced changes in program staffing or staff responsibilities. Conflicting priorities related to organizational finances or staffing levels may have also interfered with institutions’ ability to participate.

Healthy HotSpot staff provided programmatic support to community institution representatives via telephone calls, in-person meetings, and e-mails. The total number of each type of support was quantified retrospectively by accessing electronic calendars and e-mail records for the 6 Healthy HotSpot staff. Because of staff turnover and lack of telephone records, only electronic calendars (indicating phone or in-person meetings) and e-mails sent by Healthy HotSpot staff were analyzed. Data for the process evaluation included records starting March 1, 2011, through March 31, 2012, and coincided with the Healthy HotSpot initiative period from recruitment through conversion. The 8 community institutions that enrolled at least 1 corner store in the trial phase received an average of 3.4 calls, 11.8 in-person meetings, and 72.6 e-mails from Healthy HotSpot staff during this period (Table 3). The total number of Healthy HotSpot staff contacts with community institutions ranged from 62 to 118, with an average of 87.8 contacts. Healthy HotSpot staff typically did not interact directly with stores without the community institution. The 8 community institutions that enrolled at least 1 store held an average of 4.6 promotional events (range, 2–13) during the overall initiative period, including 1.8 in-store events (range, 0–5) and 2.9 community events (range, 0–8).

**Table 3:** Communication with and Training Given by Health HotSpot Staff to Community Institutions, by Municipality: Healthy HotSpot Corner Store Initiative, Suburban Cook County, Illinois, 2011–2012

Municipality	No. of Phone Calls	No. of In-Person Meetings	No. of E-mails	Total CPPW Staff Contacts	Trainings Attended
Blue Island	2	7	88	97	3
Riverdale	3	17	98	118	1
Mount Prospect	6	19	65	90	4
Calumet Park	0	11	51	62	1
Chicago Heights	3	11	89	103	1
Cicero	6	6	83	95	4
Harvey	5	17	52	74	1
Ford Heights	2	6	55	63	0
Total	27	94	581	702	NA

Abbreviation: CPPW, Communities Putting Prevention to Work; NA, not applicable.

## Interpretation

All planned aspects of the program were delivered, and more than 80% of stores entering the initiative completed both phases, thereby increasing access to healthful foods for high-need communities. There were 2 primary issues affecting success. One was delay in the implementation timeline; the other was the capacity of community institutions to carry out the expected initiative activities.

### Implementation delays

The ability to adhere to the proposed timeline was affected by several factors: 1) the short initiative time frame, which allowed little time for planning and relationship development, 2) personnel changes, 3) the process by which program documents were approved, and 4) the equipment ordering and delivery process. A community-based initiative that involves collaboration with multiple partners and municipalities requires time to develop rapport, increase the functional capacity of communities that have inadequate resources, and create sustainable change. The new process developed to recruit community institutions was successful but could have used more time to strengthen their comprehension of the initiative.

Because of the initiative structure — a county health department working with an external fiscal agent — the development of memoranda of understanding required more time than anticipated, because each institution required separate legal review. Including legal review in the initiative timeline would have eased implementation. Furthermore, the process to order, deliver, and install new store equipment was more complex and lengthy than expected. Although Healthy HotSpot staff developed an internal process to identify vendors and schedule deliveries, products were not delivered in the planned time frame. Some equipment delivery took 6 to 8 weeks, when 2 weeks had been anticipated. Discussing delivery timelines with vendors earlier could have assisted in initiative planning.

### Community institution capacity

The second factor affecting implementation was the need to increase the functional capacity of community institutions to operate independently. According to the original initiative plan, staff of the community institutions would independently perform several roles after being trained and receiving initiative materials. Those roles included store recruitment, conducting promotional events, conducting store compliance checks, and providing store support. During implementation, however, Healthy HotSpot staff was requested to help perform those functions. Only 2 community institutions did not require intensive engagement of Healthy HotSpot staff. This may have resulted from the inherent capacity of the staff members assigned at those 2 institutions or from their gained understanding of the initiative (through trainings and materials, although no consistent relationship was found between training participation and initiative success). One successful institution had a positive community reputation, experience in community health programming, and a college intern program that provided staffing throughout the initiative. That institution was able to quickly translate Healthy HotSpot initiative suggestions into action within the institution’s existing programming and structure.

In general, the ability to build on community assets, to adapt programmatic timelines to community needs, and to be inclusive of all partners’ perspectives were key to the successful conversion of 21 corner stores. In designing an initiative of this nature, it is important to consider the various administrative requirements of multiple organizations and the ability of the convening organization to quickly and adequately address challenges encountered by community partners to ensure initiative success.

Community institutions can play a key role in identifying and engaging corner stores across jurisdictions that are willing and able to implement a retail environment initiative to improve access to healthful foods in their communities. This process evaluation illustrates one effort to implement a healthy corner store initiative in a strict time frame. The complexity of institutional relationships created bottlenecks that can be anticipated during future efforts. The strengths of working with local partners included local access to stores and programming; the weaknesses resulted from lack of capacity. With its successful implementation, CCDPH has trained additional staff on this initiative and is identifying ways to build on this foundation.
